# Can Childcare Work Be Designed to Promote High Intensity Physical Activity for Improved Fitness and Health? A Proof of Concept Study of the Goldilocks Principle

**DOI:** 10.3390/ijerph17207419

**Published:** 2020-10-12

**Authors:** Anders Fritz Lerche, Maja Vilhelmsen, Kathrine Greby Schmidt, Rasmus Kildedal, Natja Launbo, Pernille Kold Munch, Mark Lidegaard, Sandra Schade Jacobsen, Charlotte Lund Rasmussen, Svend Erik Mathiassen, Leon Straker, Andreas Holtermann

**Affiliations:** 1 The National Research Centre for the Working Environment, 2100 Copenhagen, Denmark; mvi@nfa.dk (M.V.); kgs@nfa.dk (K.G.S.); rak@nfa.dk (R.K.); nla@nfa.dk (N.L.); pkm@nfa.dk (P.K.M.); ssj@nfa.dk (S.S.J.); clr@nfa.dk (C.L.R.); aho@nfa.dk (A.H.); 2 Department of Sports Science and Clinical Biomechanics, University of Southern Denmark, 5230 Odense, Denmark; 3 Novo Nordisk Health & Safety, Novo Nordisk A/S, 2880 Bagsværd, Denmark; vmlg@novonordisk.com; 4 Department of Public Health, Section of Social Medicine, University of Copenhagen, 1165 Copenhagen, Denmark; 5 Centre for Musculoskeletal Research, Department of Occupational Health Sciences and Psychology, University of Gävle, 801 76 Gävle, Sweden; SvendErik.Mathiassen@hig.se; 6 School of Physiotherapy and Exercise Science, Curtin University, Perth, WA 6102, Australia; L.Straker@curtin.edu.au

**Keywords:** physical activity, sedentary behavior, childcare workers, work environment, health promotion, workplace

## Abstract

Childcare workers are reported to have high variation in physical activity during work hours, but also to sit for about half of the workday and have almost no high intensity physical activity (HIPA). No study has investigated if their work can be re-designed to introduce HIPA, thus promoting fitness and health according to the Goldilocks principle. This study investigated the feasibility of designing pedagogical games (‘Goldilocks-games’) intended to lead to more HIPA. Heart rate was measured in nineteen childcare workers during Goldilocks-games, and compared to measurements during a regular workday. Worker perceptions of feasibility, and researcher observations of contextual factors were also collected. The Goldilocks-games (33 min) elicited significantly more HIPA (18/33 min) compared to the most active period of equal length on a regular workday (0.5/33 min). Seventy-four-percent of the childcare workers reported that it was feasible to integrate the Goldilocks-games pedagogically, and seventy-two-percent could see themselves using them. Thus, we found it possible to re-design a work task in childcare according to the Goldilocks principle so that it leads to substantial time with HIPA. The sustainability of Goldilocks-games in childcare, and their effectiveness in improving fitness and health among childcare workers, needs to be tested in further studies.

## 1. Introduction

The demographic changes and financial challenges globally require larger proportions of the population to have a longer working life [1,2]. However, low cardiorespiratory fitness [3], obesity [4], and musculoskeletal pain [5] are common health-related barriers for a long, productive working life. WHO recommends integration of health promotion in the practices and structures of the workplace to overcome such barriers and promote health of the adult population [6]. Thus, there is a need for workplaces and jobs that promote the health of workers, in contrast to wearing them down and being detrimental to their health [7]. Following the WHO definition of health as physical, mental and social well-being [8], we focus on physical activity which is a well-known positive determinant of health, as well as a protection against several chronic diseases [9,10]. Even small amounts of physical activity on a daily basis may increase cardiorespiratory fitness [11] and improve musculoskeletal fitness for an average person, and can help in maintaining the fitness level required to perform daily work tasks [9,10].

Consistent with this goal, the Goldilocks principle [12,13] argues that the fitness and health of workers should be promoted by re-designing productive work to offer a ‘just right’ variation in physical demands, for example a balance between periods of high intensity physical activity (HIPA) and recovery [12,14]. Occupations with high physical demands will likely require more recovery to achieve this ‘just right’ balance between HIPA and recovery. In contrast, sedentary occupations will need to include periods of HIPA [15]. Hence, in order to re-design work to promote fitness and health, it is necessary to retrieve knowledge about the current work situation and the workers’ health and fitness status. Subsequently, specific work activities may be manipulated in order to achieve a ‘just right’ balance [12].

Previous workplace intervention studies have investigated how work can be re-designed to increase variation by introducing more recovery [14,16]. Despite evidence for the beneficial effects of even a few minutes of HIPA per day on fitness and health [9,11], and the lack of HIPA in most jobs [17], we are not aware of any studies investigating if productive work can be re-designed to increase time with HIPA among workers.

Childcare workers have a high prevalence of poor self-rated health, obesity, musculoskeletal pain, and sickness absence [18,19,20,21]. Thus, there is a potential for improving their health through workplace initiatives focusing on health promotion. Childcare workers are reported to have large variation between physical activity behaviors at work, but also to sit for almost half of their working hours, and have almost no time with HIPA [22,23]. Thus, re-designing childcare work to promote HIPA could show a great potential for improving the cardiorespiratory fitness and health of the workers.

The European Act on Early Childhood Education and Care describes the objectives in childcare work [24]. According to this Act, an important pedagogical aim is for childcare workers to act as role models for stimulating physical activity among the children [25], and the childcare workers spend approximately 20% of their work time on activities addressing this task [26]. Some childcare research has been devoted to the physical activity of the children when playing games [27,28,29,30]. While, in these studies, the physical activity of the childcare workers would also have been of interest, no information was provided on that. A recent study found that the physical activity level of pre-school children was substantially larger if their teachers acted as role models, in the sense that they were physically active, compared to teachers who were less active [31]. We have, however, not found any studies investigating if physical activity games can be developed to induce HIPA specifically for the childcare workers themselves. Increasing HIPA as a natural part of working in childcare could help to improve cardiorespiratory fitness [11] and positively affect several health-related outcomes for childcare workers [9,10].

In a collaborative process, we therefore re-designed three games commonly used in childcare, to create ‘Goldilocks-games’, i.e., pedagogical games for children with the purpose of inducing HIPA in childcare workers. The re-design process emphasized that the new games would require the childcare workers to be physically active while also acting as role models for the children, increasing the workers physical activity.

Thus, the aims of this study were to (1) determine the extent to which the Goldilocks-games lead to HIPA for childcare workers, (2) investigate how childcare workers perceived the Goldilocks-games in regards to feasibility in daily childcare and the physical effort to perform them and, (3) identify important contextual factors when conducting the games, including participation. Our primary hypothesis was that the Goldilocks-games would lead to more time with HIPA for the childcare workers compared to a regular working day.

## 2. Materials and Methods 

### 2.1. Data Protection and Ethical Approval

The National Research Centre for the Working Environment has an institutional agreement with the Danish Data Protection Agency about procedures to treat confidential data (journal number 2015-41-4232), e.g., by securing data at a protected drive with limited access, and by anonymizing all individual data.

The Danish National Committee on Biomedical Research Ethics (The local ethical committee of Frederiksberg and Copenhagen) has evaluated a description of the study and concluded that, according to Danish law as defined in Committee Act § 2 and § 1, the intervention described should not be further reported to the local ethics committee (Ref number: H-18041423).

The reporting of this study follows the Consolidated Standards of Reporting Trials (CONSORT) 2010 statement [32]. Additionally, the intervention description follows the Template for Intervention Description and Replication (TIDieR) checklist [33]. 

### 2.2. The Process of Designing Alternative Work Tasks—The Goldilocks-Games

**Figure 1:**
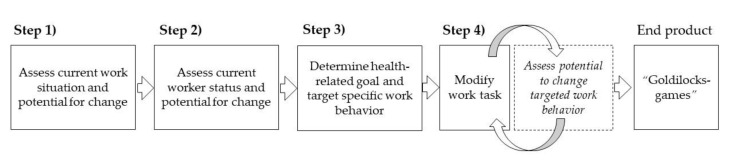
The process of designing the Goldilocks-games following the 4-steps of the Goldilocks principle.

The process of designing the Goldilocks-games is presented in Figure 1. The research team at the National Research Centre for the Working Environment was responsible for the design process of the Goldilocks-games. The process followed the procedures of the Goldilocks principle, which has been described in depth previously [12]. Briefly, the procedures comprise four steps: (1) assessing the current work situation and its potential for change, (2) assessing the status of the workers and their potential for change, (3) specifying health-related goal(s), and (4) reorganizing or modifying current work tasks according to the Goldilocks principle to meet the goal(s) [12].

Step 1: We retrieved evidence from two childcare institutions participating in a previous study by our research group [23]. Based on this knowledge and a dialogue with childcare workers and managers, we identified what constituted a regular workday, including identifying main work tasks, and where different activities occurred. This gave an overview of childcare work and an idea of the potential to achieve a ‘just right’ balance of exertion and recovery.
Step 2: We collected additional data on 15 childcare workers (age, *M* 38 years, *SD* 11; 60% female) at the two institutions engaged in step 1. Data was collected regarding BMI (*M* 26 kg/m^2^, *SD* 4), self-reported physical activity level, and heart rate for five consecutive days, the latter using ambulatory sensors. This provided a basis to assess the workers’ health status, and physical behavior(s) that could influence health.
Step 3: Based on our understanding of the childcare workers’ (i) work tasks, (ii) physical activity behavior during work hours, and (iii) health status, we determined that a relevant health-related goal would be to improve their cardiorespiratory fitness. To achieve this goal, we focused on promoting HIPA behavior when the childcare workers play pedagogical games with the children.
Step 4: The process of modifying pedagogical games in childcare began by identifying initiatives that could increase HIPA in childcare, based on scientific literature [34,35], field observations and feedback from childcare workers. Then, an iterative process (shown by circulating arrows, Figure 1) was used to develop pedagogical games (i.e., modify a work task) and test them in daily childcare work (i.e., assessing their potential to change work behaviors). The field tests were performed on four childcare workers at two separate institutions, none of which were included in the main study. The field tests included observations, questionnaires and measurements using ambulatory sensors to assess the extent to which the games lead to HIPA, and whether it was feasible for the childcare workers to perform the games in the context of acting as active role models for the children. In this process, we developed and tested six games. Three of the six games did not show a potential to increase HIPA, and were therefore excluded. Finally, we presented the three remaining games to an expert panel comprising members from childcare organizations, union representatives, employers associations’ representatives and government occupational health consultants, which added specific suggestions on how to tailor the games to the needs, context, and work conditions in childcare. This included adapting the games to better incorporate *the new and improved educational curriculum* (i.e., six politically determined objectives) for childcare work in Denmark [24].

The final outcome of the design process was, thus, three alternative work tasks termed ‘Goldilocks-games’: (1) Egg Hunt, (2) The Goldilocks Train, and (3) The Rabbit Hunt. All games were re-designed to secure that the childcare workers would act as role models by being physically active, e.g., by walking fast, running, or jumping, which would in turn, lead to HIPA for the workers themselves. Thus, the rules of the original three games were modified so that all games now required the workers to move. All games were interval-based (i.e., short intense bouts and rest breaks) and gave the children a physical advantage (e.g., a head start). In some games, workers had to catch the children; in one game, workers did additional physical activities to raise their heart rate, before even involving the children. Modifications made to each game along with a description of each game is provided in detail in Appendix A and Appendix B, respectively. The Egg Hunt and The Rabbit Hunt games were different versions of the game ‘tag’, focusing on the childcare workers needing to catch the children as well as doing full body exercises, e.g., high knees or ‘jumping jacks’. The Goldilocks Train game was an interval-based activity where the children held hands in pairs and formed a constantly moving line (i.e., the ‘train’). The childcare workers then had to bring children from the back of the train to the front, as fast as possible, by walking fast or running. 

### 2.3. Study Design

**Figure 2:**
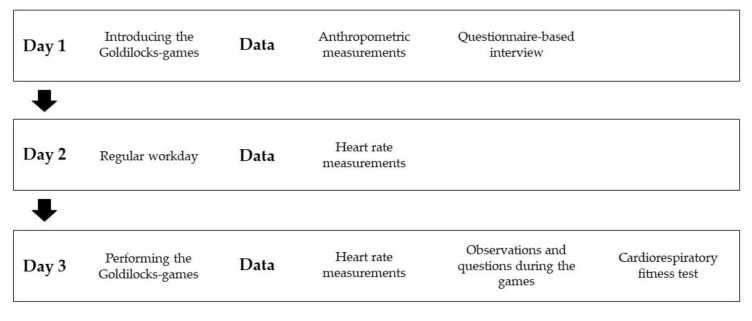
Study design for evaluating time with HIPA during a regular workday and the Goldilocks-games.

Figure 2 provides an overview of the three-day study design used to further investigate the three Goldilocks-games described above. Data was collected between October 2019 and February 2020. In Denmark, childcare workers are typically organized in teams of two to three, being responsible for a group of 12 to 18 children. Therefore, the participating childcare workers worked in pairs, which were observed for one three-day period each (Figure 2). Twenty-two childcare workers completed the data collection period, and thus data collection was performed 11 times in total.

Day 1: Began with anthropometric measurements and a questionnaire-based interview with the childcare workers. After that, we instructed the children to play the Goldilocks-games with the childcare workers joining in together with the children, thus introducing the childcare workers to the games.
Day 2: A regular workday, where the childcare workers were told to perform their work as usual.
Day 3: Began with the childcare workers conducting the Goldilocks-games with the children without assistance from the research team. A member of the research team observed the Goldilocks-games and noted information on participation by the childcare workers, and contextual factors of relevance. Also, the childcare workers were asked to rate their perceived exertion while performing the games. After having performed the games, the childcare workers were asked about the feasibility of the games, and had their cardiorespiratory fitness assessed. Heart rate was monitored during day (2) and (3).

### 2.4. Participants

Childcare workers (*n* = 22) were recruited from public childcare institutions located in the greater Copenhagen area in collaboration with the local municipalities. The study was announced through e-mails distributed to the childcare institution managers, who in turn asked the childcare workers to participate in the study. For strategic reasons, we decided that all institutions expressing interest in the study were eligible to participate, even though this would likely result in a study size substantially larger than needed according to the power analysis. Inclusion criteria for individual workers required them to primarily work with children two to five years of age. Exclusion criteria were: pregnancy, adhesive tape allergy, fever on the day of anthropometric measurements, and use of pacemaker. All participants signed a written informed consent prior to participating in the study. The childcare workers participated in pairs during days when the Goldilocks-games were tested, without any criteria for pairing.

We collected self-reported information on age, sex, job title, current length of service, current smoking habits, working hours per week, and time per week spent with moderate and vigorous physical activity (MVPA), including how much of that time was spent exclusively with vigorous physical activity (VPA).

Anthropometric measurements of body height [cm], weight [kg], body mass index (weight [kg]/ (height squared [m^2^])), fat percentage (BC-418 MA body composition analyzer; Tanita, Tokyo, Japan), and resting blood pressure (Omron M3 r Omron M6 Comfort; Omron Corporation, Kyoto, Japan) were also collected. An assessment of cardiorespiratory fitness was conducted on day 3 at least 10 min after finishing the Goldilocks-games using the Ekblom Bak submaximal cycle ergometer test (Monark AB, Varberg, Sweden). This test has shown good validity among a wide range of adults [36] and was found suitable for the present study as it can easily be carried out at most workplaces, has a reasonably short duration (8–10 min), and is likely better accepted and performed by employees less experienced with HIPA than tests performed at higher intensities. Cardiorespiratory fitness (VO_2max_) was estimated based on the difference in heart rate between an initial low standard workload and a subsequent higher ‘final’ workload [37]. Heart rate during the ergometer test was monitored using an arm-worn Polar^®^ OH1 (Polar Electro Oy, Kempele, Finland) heart rate monitor, which has been shown to provide accurate heart rate data [38,39]. A worker’s fitness level was classified into one of three categories; “below average”, “average” and “above average”. The classification was based on categories used with the Ekblom Bak test [40], thus the scores “very low” and “low” was pooled into “below average”, the scores “somewhat low”, “average” and “somewhat high” was pooled into “average” and lastly the scores “high” and “very high” was pooled into “above average”. The three categories correspond to the 0–25, 25–75, and 75–100 percentiles of the reference population, respectively [40]. 

### 2.5. Worker Perceptions of Feasibility and Exertion

Twenty-two childcare workers performed all three Goldilocks-games together in pairs, forming a team with the children. Thus, the games were performed on eleven different occasions. Immediately following each Goldilocks-game, all participating childcare workers were asked to judge to what extent that Goldilocks-game was (1) feasible to conduct as a part of the pedagogical work and (2) feasible to implement in their daily work in the future. Both questions were answered using a 5-point Likert scale ranging from “to a very low degree” to “to a very high degree”. We converted the 5-point Likert scale answers into the game being either feasible (the top two answers), partially feasible (the middle answer), or unfeasible (the bottom two answers). Eventually, feasibility was summarized in terms of the percentage of answers falling into each of the three categories, all three Goldilocks-games combined. Workers also rated their physical exertion on a Borg scale from 1 to 10 [41], and we calculated a mean score for the three games for each individual worker.

### 2.6. Participation and Contextual Factors 

During the eleven occasions when the three Goldilocks-games were performed, a member of the research team observed and noted, (1) the time and total duration of the three games, (2) contextual factors, i.e., location, access to and size of location, weather conditions, and interruptions, (3) the number of children participating, number of children leaving the game, and number of childcare workers leaving for more than half of the game, (4) number of adjustments made by the childcare workers to the games (e.g., changing the rules of a game), and (5) the level of physical activity among the children and childcare workers during each game (i.e., a visual judgement of movements and/ or signs of exhaustion), rated by the observer on a 5 point Likert scale from “a very low extent” to “a very high extent”. The 5 point Likert ratings of the children’s physical activity level was averaged for all three games and counted as one combined score for each occurrence of the games (*n* = 11). As data on contextual factors consisted of several uncategorized textual descriptions, we determined a set of categories compromising weather conditions (sunny, raining, or cloudy), area size (>100 m^2^ or <100 m^2^), and type of place (soccer field, park, or playground). Data on the physical activity level among childcare workers and children was averaged across the three Goldilocks-games for each childcare worker and group of children. 

### 2.7. Heart Rate 

To enable accurate assessment of heart rate reserve (HRR), Firstbeat Bodyguard 2 heart rate monitors (Firstbeat Technologies Ltd., Jyväskylä, Finland) were mounted on day one, placing Ag/AgCl pre-gelled electrodes below the right clavicle and at the left rib cage. The childcare workers were asked to wear the heart rate monitor around the clock during all three days and were instructed how to change the electrode tape if necessary. After measuring for three days, data was downloaded using the Firstbeat Uploader software (Firstbeat Uploader Version 3.1.2.0; Firstbeat Technologies Ltd.) and further processed for evaluation of beat errors using an established software [42,43]. A heart rate recording was excluded from further analysis if the beat-error was ≥50% [17,42,44].

The intensity of the physical activities was estimated by %HRR, i.e., the difference between the estimated maximal heart rate and the resting heart rate, according to the equation proposed by Tanaka et al. [45]. Resting heart rate was defined as the lowest recorded heart rate value during the first night’s sleep, consistent with other studies of %HRR during work [17,44]. Occurrence of HIPA was expressed in terms of time ≥60%HRR, since intensities above this threshold have been suggested to be effective in improving cardiovascular fitness [46,47]. 

During all three measurement days, the childcare workers were requested to note in a diary at what time they, (1) woke up, (2) arrived at work, (3) left work, (4) went to sleep, and/or (5) if the heart rate monitor was detached. A member of the research team noted the time when the three Goldilocks-games were performed. On basis of the diaries and observations, the continuous timeline of %HRR data was partitioned into periods of, (1) working hours without Goldilocks-games, (2) Goldilocks-games, (3) leisure time awake, and (4) sleep.

### 2.8. Sample Size

A necessary sample size was determined a priori in a power calculation based on heart rate data collected in the design process of the Goldilocks-games (step 1, cf. Section 2.2) from seven childcare workers over five consecutive days in a regular workweek. The arithmetic mean value of time in HIPA, i.e., ≥60%HRR, was 1.7 min, with a *SD* of 2.5. *SD*, this value was post-hoc adjusted due to fit an expected higher *SD* when performing Goldilocks-games, arriving at a 3 times larger estimate (i.e., *SD* 7.5). The desired minimal detectable difference in HIPA between a regular workday and the Goldilocks-games day was set to 10 min at ≥60%HRR. A repeated-measures design having a power of 0.80 in detecting this difference at a significance level of 0.05 would require nine childcare workers. Assuming a drop-out of 20%, a study would, thus, require at least 11 participants [48]. In selecting 10 min as the minimum detectable effect size, we chose an amount of HIPA that would lead to improved cardiovascular fitness [11] and positively contribute to several health-related outcomes for the average subject if performed for five days a week [9,47,49,50].

### 2.9. Statistical Methods

All descriptive data was checked for normal distribution and outliers using histograms and visual inspection of normality diagrams. All numerical data is summarized by the mean and *SD* across workers. To describe the distribution of time spent at different intensities during the Goldilocks-games more in detail, we calculated arithmetic means of time spent in the following %HRR intervals: 0–60, 60–70, 70–80, 80–90, and 90–100.

**Figure 3:**
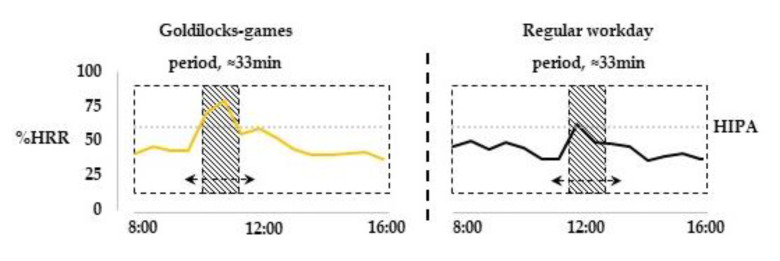
Illustration of the procedure used to compare time spent with HIPA during the Goldilocks-games and during a regular workday. The shaded area shows the periods compared with occurrence of HIPA.

To address the primary hypothesis, i.e., the extent of HIPA during Goldilocks-games compared to regular work, we identified in a regular workday, for each participating worker, the most active period (i.e., the period comprising the most HIPA) of the same duration as the Goldilocks-games (Figure 3). Active play with the children is a common task in regular childcare, occupying around 45–60 min every day [26]. However, we did not record the exact time of the scheduled active play on the regular workday (cf. Figure 2), and so we decided to locate the ‘most active’ period, as identified by having the maximum HIPA time within a moving window of a duration corresponding to the Goldilocks-games for that specific worker. Time spent at ≥60%HRR and <60%HRR was then calculated for the periods with Goldilocks-games and regular work, both in terms of minutes and in terms of percent time (Figure 3).

Further processing and comparisons were done using compositional data analysis [51,52]. Using procedures in Rstudio v. 1.2.5033 [51] and the *compositions* [53] package, we expressed the central tendency of the data in terms of the geometric means of time ≥60%HRR and <60%HRR for the two periods [54]. These compositional means were then normalized to either 33 min (i.e., the average duration of heart rate measurements) or 100%.

We then compared the 2-part compositions (≥60% HRR vs <60% HRR) between the regular workday period and the Goldilocks-games period, using isometric log-ratio [55] coordinates in a repeated-measures ANOVA.

Two workers spent zero time ≥60% HRR in the regular workday period. These zero observations were assumed to be due to too little measurement data during working hours, and thus they were considered rounded zeros [56]. We used the *zCompositions* [57] package to replace the zeros by expected values based on the information in the covariance structure of the observed data using a log-ratio Expectation-Maximization algorithm [58]. The primary analysis comparing Goldilocks-games and regular work included these two workers with imputed heart rate data, and to verify the robustness of the results, we also conducted a sensitivity analysis excluding the two workers.

Descriptive statistics were used to address the second and third study aims. Worker perception of feasibility measured on an ordinal scale is presented as mean percentages for the group. Perceived exertion was rated on an interval scale and is summarized as the group mean (with SD) of each worker’s mean rating across the three Goldilocks-games. Researcher observations of participation and contextual factors is reported for all occurrences of Goldilocks-games and is presented on a nominal scale as frequency (N) and percentage, or, for numerical data, as a group mean and SD.

## 3. Results

### 3.1. Participants Flow

**Figure 4:**
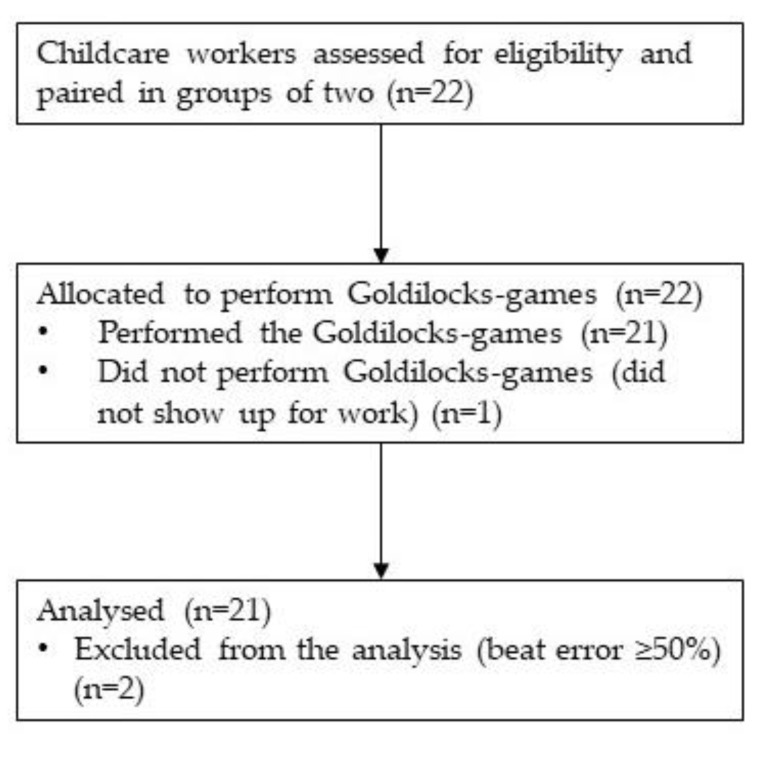
Participant flow, leading to the final sample of 19 workers.

Childcare workers from five childcare institutions were enrolled in the study (*n* = 22; Figure 4). On one occasion, a participating childcare worker did not show up for the games and was therefore substituted by a colleague who was not wearing a heart rate monitor during the games. Further, two workers had a beat error ≥50% and were excluded. The excluded childcare workers were not on the same team. Thus, data for analysis of primary and secondary outcomes were available from nineteen workers (*n* = 19) participating at one of the eleven (*n* = 11) occasions when the three Goldilocks-games were performed.

### 3.2. Demographics and Fitness

The childcare workers were predominantly female (68.4%), middle-aged (*M* 35.3 years, *SD* 11.5), and had a wide range of work experience (Table 1). The workers reported spending about six hours weekly performing MVPA during work and leisure combined, including one-and-a-half hour during work, and that two of those hours were spent in VPA. Participants were generally overweight (BMI: *M* 25.0 kg/m^2^, *SD* 3.6), had a normal blood pressure, and an ‘average’ fitness level (63.2%).

### 3.3. Worker Perceptions of Goldilocks-Games Feasibility and Exertion

All childcare workers perceived the games to be either feasible or partly feasible to be included as part of the pedagogical work, and only 7.0% reported that it was not feasible to implement the games in their daily work in the future (Table 2). On average, workers rated their exertion during the games as 5 on the Borg scale, i.e., ‘severe’.

### 3.4. Researcher Observations of Contextual Factors during the Goldilocks-Games

All Goldilocks-games were conducted outdoors (100%) and most often in a large area >100 m^2^ (72.7% of the games; Table 3).

On average, the Goldilocks-games lasted around half an hour (*M* 32.5 min with valid HR data, *SD* 8.1) and included around a dozen children (*M* 13.0 participating children, *SD* 4.4). Few children left during the games (*M* 1.4 children leaving, *SD* 2.1), and only once was a childcare worker absent for more than half a game. Most of the games were performed without any external disturbances, e.g., other children interfering, or children requiring acute attention elsewhere from the participating childcare workers. Only few adjustments were made to the games by the childcare workers and we observed that both children and workers were moderately to highly active during the games.

### 3.5. Heart Rate Measurements (HIPA during Goldilocks-Games and Regular Workday)

**Figure 5:**
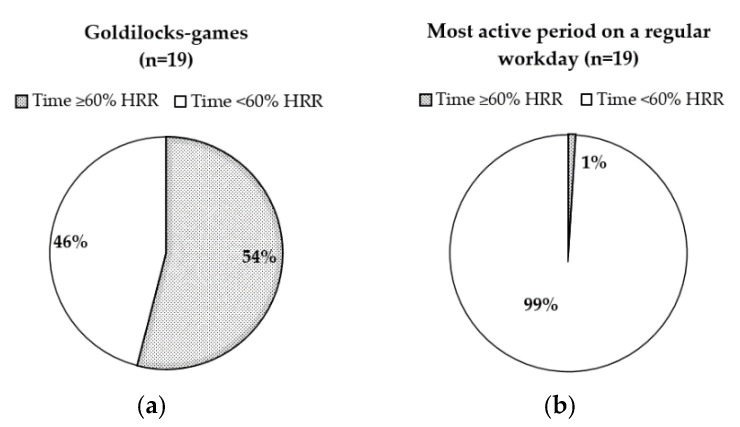
Compositions (geometric means) of time (in terms of percent) spent with and without HIPA, (**a**) during the Goldilocks-games, (**b**) during the regular workday period. Abbreviations: HRR, heart rate reserve.

During their most active period of the regular workday, the childcare workers spent (geometric mean) 0.3 min (1% of the time) at ≥60% HRR, while 17.8 min (54%time) were spent at ≥60% HRR during the Goldilocks-games (Figure 5). The difference between the two compositions was statistically significant (*p* < 0.001). The difference was still highly significant when excluding the two workers with imputed HRR data.

**Figure 6:**
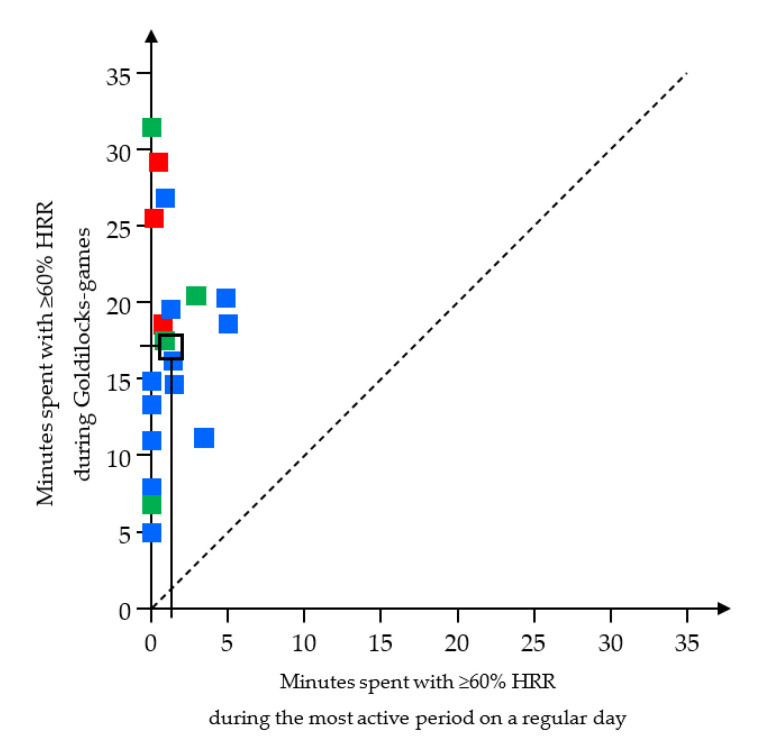
Minutes spent in HIPA (≥60% HRR) during the most active period in a regular workday (x-axis), and during Goldilocks-games (y-axis). Red, blue and green symbols show values for each individual childcare worker in the ‘below average’, ‘average’ and ‘above average’ fitness groups, respectively; the black square (with vertical and horizontal lines) marks the arithmetic mean of the entire group (*n* = 19). The dashed line illustrates the line of identity. Abbreviations: HRR, heart rate reserve.

All of the childcare workers achieved more HIPA during the Goldilocks-games than they did during the most active period on a regular workday (Figure 6).

**Figure 7:**
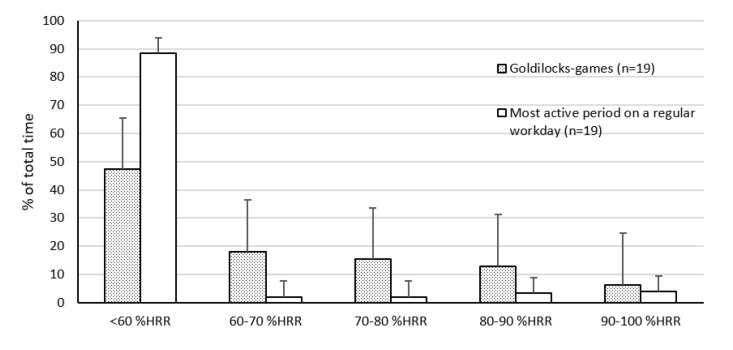
Distribution, in terms of percent time, of percent heart rate reserve during the Goldilocks-games (grey bars) and during the most active period on a regular workday (white bars). Bars show arithmetic group means, with *SD* between workers illustrated by the error bars. Abbreviation: HRR, heart rate reserve.

Further, 16 out of 19 workers achieved at least 10 min with HIPA during the Goldilocks-games, while none of the workers did so during the most active period on a regular workday (Figure 6). Exploratory analysis showed that the three fitness groups appeared to differ slightly in HIPA time when performing the Goldilocks-games (below average fitness 24.5 min, average fitness 14.9 min, above average fitness 19.1 min; individual results in Figure 6), but the limited number of participants in some groups precluded inferential testing. Arithmetic means of the heart rate distribution during Goldilocks-games are presented in Figure 7. During the Goldilocks-games, the childcare workers had 15.1 min (*SD 7.1*, *47.2%*) with 0–60%HRR, 5.8 min (*SD 2.1*, *18.0%*) with 60–70%HRR, 5.0 min (*SD 2.1*, *15.3%*) with 70–80%HRR, 4.4 min (*SD 3.6*, *13%*) with 80–90%HRR, and 2.2 min (*SD 3.0*, *6.4%*) with 90–100% HRR.

Additional exploration was conducted to examine perceptions of feasibility and exertion between the three fitness groups, and between smokers and non-smokers (See Appendix C and Appendix D). Participants were again too few for making inferences, but we observed a tendency for less fit workers to rate their exertion higher, and for smokers to be more reluctant regarding the feasibility of the games.

## 4. Discussion

The main finding of this study was that it is feasible to introduce more HIPA among childcare workers by re-designing work tasks according to the Goldilocks principle. Thus, the studied workers spent about 18 min of the 33 Goldilocks-games minutes in HIPA (i.e., 54% of the time), compared to only 30 sec (i.e., 1% of the time) during the most active period on a regular workday.

To our knowledge, this is the first study to develop and test work tasks re-designed according to the Goldilocks principle in childcare. Previous studies in childcare have investigated how to modify games to increase MVPA in children [30], promote physical activity in general among the children [28,29], and reduce physical work demands among childcare workers [59], but no study has investigated whether work task(s) in childcare can be successfully re-designed to lead to more HIPA among the childcare workers. Overall, utilising the 4-step procedure of the Goldilocks principle was feasible in childcare work; assessing the current work situation and potential for change, assessing the current health status of the workers and the potential for change, specifying a health-related goal and target a behavior at work, and finally, re-designing a work task to meet the goal [12].

**Figure 8:**
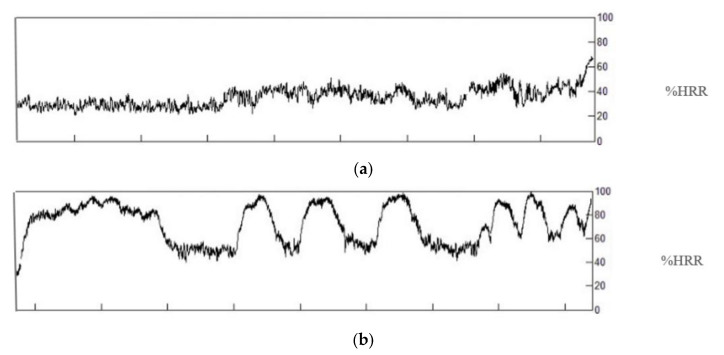
Representative example of one worker’s heart rate; (**a**) during the most active period on a regular day and; (**b**) during the three Goldilocks-games. Abbreviation: HRR, heart rate reserve.

The developed Goldilocks-games led to substantially more time with HIPA compared to the period in the regular workday with most HIPA. The observed duration of HIPA during normal childcare work is unlikely to promote fitness and health. Figure 8 shows the variation in %HRR during the three games, which result in three intervals with high intensity, i.e., one per game. Even short durations of HIPA during work hours have been shown to significantly improve cardiorespiratory fitness [60]. Also, Gillen et al. [11] showed that three cycling bouts of twenty seconds each at maximal intensity (i.e., one minute in total), performed within a ten minute period, resulted in similar effects on fitness, and indicators of muscular health, as efforts of moderate intensity performed for 50 min. On average, the Goldilocks-games lead to two minutes at 90–100%HRR within a 30 min period. Thus, they can be scheduled as a time-efficient work task with a significant effect on cardiorespiratory fitness and, likely, health [49,61]. If, for instance, the Goldilocks-games can be performed three times a week as part of the ordinary routine in childcare, this could have positive health effects in most childcare workers, with a potential to prevent several chronic diseases (e.g., cardiovascular disease and type 2 diabetes) and premature mortality [9,10].

Feasibility was confirmed by the childcare workers, predominantly reporting the Goldilocks-games to be feasible to conduct as a part of the pedagogical work for the children (75% of the workers) and to be implementable in their daily work (71%). An essential part of the Goldilocks principle is that re-designed work tasks must not compromise productivity. In childcare, the core tasks are care for, play with, and educate children. Therefore, it was imperative to investigate if the childcare workers found that the Goldilocks-games could be implemented as a natural part of their pedagogical work. We received strong indications from the childcare workers that the Goldilocks-games were both relevant to their pedagogical work and desirable to use. Further, the children were observed to have moderate to high physical activity levels during the Goldilocks-games, suggesting that the games were effective even in promoting physical activity for the children. High intensity physical activity in children has been shown to positively affect cardiometabolic risk markers [62], suggesting that, playing the games could also benefit the children’s health. The children’s observed physical activity level during the Goldilocks-games is consistent with a recent study reporting an increased physical activity level among children when teachers acted as physically active role models [31]. The low number of children leaving the games (typically 1 or 2 children out of an average of 13 participating children per Goldilocks-game) indicates that the Goldilocks-games were acceptable even according to the children. Together, these results suggest that the Goldilocks-games are consistent with the pedagogical work of childcare workers.

The childcare workers were able to achieve HIPA at a rated perceived exertion of no more than five out of 10. This is similar to exertion ratings for recreational football, jogging, or heavy gardening [63,64]. Being able to achieve HIPA at a not-too-high exertion in childcare is important to the eventual feasibility of the Goldilocks-games, since the workers need to have sufficient energy during the games and for the rest of the day to manage and care for the children.

We found that the Goldilocks-games were almost always conducted in a large area outdoors (8 out of 11 sessions in a large area, all of them outdoors). This is consistent with other studies, finding that a large available area, and being outdoors facilitates physical activity in childcare [34,35]. In order to find a suitable large area, eight out of the 11 game sessions were conducted at a facility away from the institution. All participating institutions were located in a densely populated area close to large roads and commuting traffic. Consequently, going to an external facility that would fit the criteria of having a large area size and being outdoors could mean crossing large roads with heavy traffic, which may be an important barrier for performing Goldilocks-games in some cases.

### 4.1. Strengths and Limitations

A strength of this study was the collaborative approach used in the design process of the Goldilocks-games [65]. Involving many different stakeholders, such as childcare workers, managers, professional experts, and union representatives, secured that many relevant stakeholders within childcare contributed to design the Goldilocks-games. Further, the iterative process of modifying and testing the games with childcare workers and children contributed to securing the pedagogical content and the feasibility of performing the games, and thus their sustainability in childcare.

Another strength was the use of accurate heart rate measurements [66] to determine the relative physiological intensity (i.e., % HRR) [67] of physical activity accumulated during the childcare tasks.

Several limitations should, however, be noted when interpreting the results of this study. First, we excluded the heart rate data from two workers due to a high beat error (≥50%). The main reason for the high beat error was most likely rapid body movements disturbing the signal of the heart rate sensor (i.e., motion artefact) during the games. Thus, we suspect that mainly periods with HIPA would be missing from these measurements, and that this would likely affect both the regular work day and the Goldilocks-games equally.

The voluntary recruitment process could have led to selection bias. We don’t know if only the most motivated kindergartens and childcare workers participated. Further, we found that the participating workers were healthier, more physically active, and had a higher cardiorespiratory fitness level than expected. Therefore, their positive inclination towards engaging in games leading to HIPA may not be generalizable to childcare workers and institutions in general.

The study design implied that the researchers introducing the childcare workers to the games were also present during the data collection. This could have introduced a participant bias in that the workers unintentionally behaved and answered questionnaires differently, reporting higher feasibility scores and being more inclined to perform HIPA. In extension, implementation of Goldilocks-games in childcare should be evaluated in larger trials, which assess sustainability and measure health effects of the games on a longer term. Thus, a randomised controlled trial among Danish childcare institutions has been initiated [68].

### 4.2. Practical Implications

According to the results of our study, applying the Goldilocks principle in a childcare setting could have a sustained beneficial impact for both childcare workers, childcare institutions and the children. Implementing the Goldilocks-games in childcare introduces HIPA among childcare workers by re-designing a common work task in childcare (playing with the children), which also contributes to an important educational curriculum. Since the Goldilocks-games can be implemented as a part of productive work in childcare and contribute to realizing important pedagogical aims, it should be possible to schedule them as a strategic work task. In extension, that may even overcome challenges associated with ordinary recommendations for performing HIPA after work, such as the worker being tired at the end of the day, having limited time, and facing a number of competing after-work responsibilities [69]. Furthermore, the HIPA achieved in this study could promote fitness and health of the childcare workers, which could, in turn, potentially reduce sickness absence and, thus, costs for the childcare institution [70]. In order to make the Goldilocks-games generally accessible to Danish childcare workers not involved in the present study, we are currently developing materials that can guide workers into conducting Goldilocks-games, and assist them in creating their own games.

Re-designing productive work to include beneficial physical activity could reach otherwise inactive, unfit, and overweight employees not being active during their leisure time, with potentially positive effect on their fitness and health. Failure of reaching the most inactive workers has previously been identified as a shortcoming of initiatives focusing on health promotion at the workplace [71].

## 5. Conclusions

HIPA is almost absent in regular childcare work. The main finding of this study was that a re-designed work task, the Goldilocks-games, introduced substantial HIPA into the work, which could likely promote fitness and health among the workers. The majority of the childcare workers considered the Goldilocks-games to be pedagogically relevant, and usable as part of their daily work. Together, these findings suggest that Goldilocks-games could be a feasible and time-efficient way to introduce substantial HIPA into childcare, for the purpose of promoting the fitness and health of workers, in accordance with the Goldilocks principle.

## Figures and Tables

**Table 1:** Demographic and fitness characteristics of the childcare workers (*n* = 19).

Variables	*N*	%	Mean (*SD*)
Sex (*female*)	13	68.4	
Age (*years*)			35.3 (11.5)
Length of service in current job			
*<3 months*	8	42.1	
*12–120 months*	9	47.4	
*≥120 months*	2	10.5	
Self-rated time spent in MVPA during work and leisure combined (*hours/week*)			6.2 (4.4)
Self-rated time spent in MVPA during work (*hours/week*)			1.4 (2.5)
Self-rated time spent performing VPA during work and leisure (*hours/week*)			1.9 (1.7)
Current smoker (*yes*)	7	36.8	
BMI *(kg/m^2^)*			25.0 (3.6)
Blood pressure *(mmHg)*			
*Systolic*			124.9 (10.3)
*Diastolic*			81.8 (8.4)
Cardiorespiratory fitness *(ml/kg·min)*			41.9 (7.9)
*Below average*	3	15.8	
*Average*	12	63.2	
*Above average*	4	21.1	

Values are frequency (*N*), percentage (%) or mean (*SD*).

**Table 2:** Worker perceptions of Goldilocks-games feasibility and exertion (*n* = 19).

Variables	%	Mean (*SD*)
Pedagogic feasibility ^a^		
*Unfeasible*	0.0	
*Partially feasible*	26.3	
*Feasible*	73.7	
Future implementation ^b^		
*Unfeasible*	7.0	
*Partially feasible*	21.1	
*Feasible*	71.9	
Perceived physical exertion ^c^		5 (1.4)

Values are percentage (%) or mean (*SD*).; ^a^ “To what extent is the game usable in the pedagogical work?”.; ^b^ ”To what extent can you see yourself perform the games in your daily work?”.; ^c^ Borg CR10 scale (1 to 10).

**Table 3:** Researcher observations during the Goldilocks-games (*N* = 11 occasions).

Contextual Factors	*N*	%	Mean (*SD*)
Location ^a^			
*Indoor*	0	0.0	
*Outdoor*	11	100.0	
Area ^b^			
*Football field*	4	36.4	
*Playground*	0	0.0	
*Park*	7	63.6	
Area size ^c^			
*>100 m^2^*	8	72.7	
*<100 m^2^*	3	27.3	
Facilities ^d^			
*On institutions facilities*	3	27.3	
*On other facilities*	8	72.7	
Easy access ^e^			
*Yes*	9	81.8	
*No*	2	8.2	
Weather ^f^			
*Sunny*	4	36.4	
*Cloudy*	6	54.6	
*Raining*	1	9.0	
Duration (min)			32.5 (8.1)
Children participating			13 (4.4)
Children leaving			1.5 (2.1)
Workers’ leaving ^g^			
*Yes*	1	9.1	
*No*	10	90.9	
Interruptions ^h^			
*Yes*	2	18.2	
*No*	9	81.8	
Adjustments ^i^			1.3 (0.8)
Area limitations ^j^			
*Yes*	3	27.3	
*No*	8	72.7	
Physical activity among the children ^k^			
*High or very high*	3	27.3	
*Moderate*	8	72.7	
*Low or very low*	0	0.0	
Physical activity among the workers ^l^			
*High or very high*	7	63.6	
*Moderate*	4	36.7	
*Low or very low*	0	0.0	

Values are presented as frequency (*N*), percentages (%) or mean (*SD*). ^a^ ”Where were the games conducted, inside or outside?”; ^b^ ”At what specific area were the games conducted?”; ^c^ ”State the area size”^; d^ ”Were the games conducted on the institutions facilities?”; ^e^ ”Was there easy access to the area?”; ^f^ ”Describe the weather conditions”; ^g^ ”Did any Childcare worker leave for more than 50% of the game duration”; ^h^ ”Were the childcare workers interrupted during the games?” (e.g., children outside the game interfering, or requiring acute attention); ^i^ ”Were the games adjusted by the childcare workers during play?” (i.e., not strictly following the guide description); ^j^ ”Was the area not useful for conducting the games?” (e.g., too small, too many obstacles etc.); ^k^ ”To what extent were the children physically active during play?”; ^l^ ”To what extent were the childcare workers physically active during play?”.

**Figure A1:**
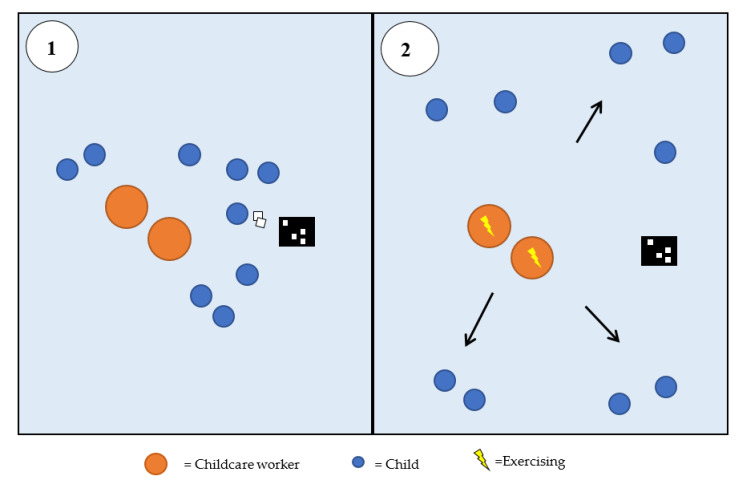
The Egg hunt. Further details in the text.

**Figure A2:**
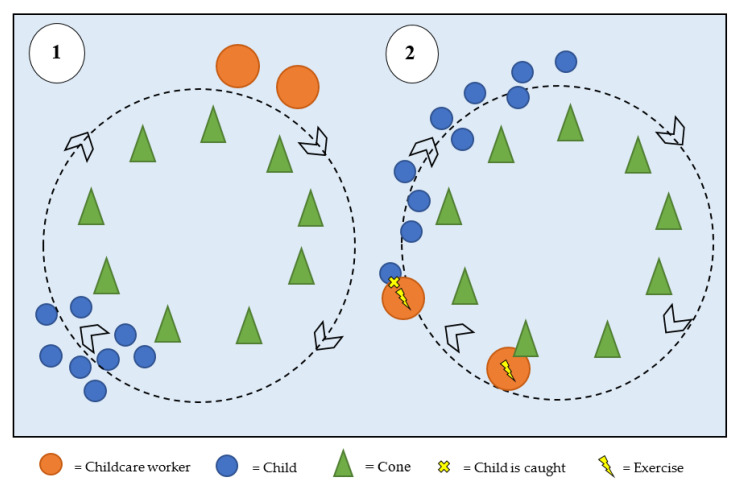
The Rabbit hunt. Further details in the text.

**Figure A3:**
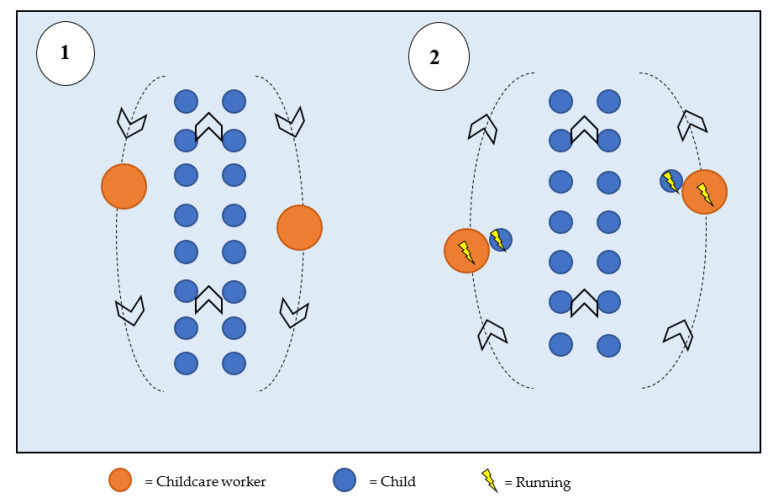
The Goldilocks Train. Further details in the text.

## Appendix A

**Table A1:** Description of games/activities and Goldilocks principle modifications.

Original Game	Description	Re-Designed Game	Modifications
Catching tails	Two children are randomly announced as catchers. The remaining children each attach one tail. The catchers countdown from ten and then run to collect all the tails from the children. The childcare workers assist in attaching tails and start and stop the game.	Egg hunt ^1,2,3,4,5^	The childcare workers are announced as catchers ^1^. All the children attach eggs to their shirts/pants. The childcare workers draw an activity card and countdown by performing 1–9 repetitions of the drawn activity (e.g., burpees, air boxing or air squats), while the children runs away ^2,3^. The childcare workers then have to collect all the eggs by chasing and catching each child ^1^. The game is restarts for several rounds ^4^.
Circle run	Two equal sized teams of children starts at opposing sides of a circle of cones. The objective is to catch the opposing team by running in the same direction around the circle. The childcare workers set up the circle, judge who is caught and restarts the game.	Rabbit hunt ^1,2,3,4,5^	The childcare workers and a group of children starts at opposing sides of a circle of cones. The childcare workers have to catch the children running in the same direction as the children around the circle ^1^. When a child is caught, the childcare workers perform an activity (e.g., burpee, air boxing, air squats), giving the remaining children the opportunity to run forward ^2,3^. When all children are caught the game restarts ^4^.
Train	All children are paired and walks hand-in-hand in a straight line. The childcare workers walks at the front and the end of the line.	Goldilocks train ^1,3,4,5^	All children are paired and walks hand-in-hand in a straight line. The childcare workers walks in the front. The childcare workers walk to the back of the line, and collects one child each from their side of the line ^1,3^. The childcare worker and the child (holding hands) run together to the front of the line ^1^. This process is continued until a predetermined destination is reached ^4^.

^1^ The game requires the childcare worker to be physically active. ^2^ The childcare workers did additional physical activities to raise their heart rate as part of initiating the game. ^3^ The children gets a physical advantage over the childcare workers. ^4^ Interval-based game setup. ^5^ Few and simple rules (apply to all re-designed games).

## Appendix B

### Appendix B.1. Game 1: Egg hunt

All Children gets a laminated egg attached to their shirt with a clamp. A child then draws an exercise card and a number card (i.e., 4–9). The childcare workers then performs the number of repetitions from the number card of the drawn exercise, this is the countdown. Meanwhile the children gets a head start and run away from the childcare workers. When the countdown is finished, the childcare workers should collect all the eggs from the children. When all eggs are collected the game is finished and will restart. The game continues for 3–4 rounds, or until 10–15 min has passed.

- Exercises: Burpees, squat jumps, jumping jacks, air boxing with high knees.
- Materials: Laminated eggs, clamps, exercise and number cards.
- Duration: 10–15 min.

### Appendix B.2. Game 2: The Rabbit Hunt

Eight to twelve cones are placed so they form a large circle.

The childcare workers is acting as the fox (the catcher). The children are the rabbits, and should avoid getting caught by the foxes. Foxes and rabbits is placed on the opposite end of the circle and it is only allowed to run in the same direction around the circle.

The game begins with the foxes chasing the rabbits. When a fox catches a rabbit all foxes should do an exercise (i.e., burpees, jumping jacks etc.) in order to give the rest of the rabbits a head start. After the exercise is performed the foxes can again catch the rabbits. The first three round are played in this manner. On the fourth round, foxes and rabbits switch roles (rabbits has to catch the foxes). On the fifth round, they switch back (foxes has to catch the rabbits) and now the foxes has to catch all the children. The game ends after all rabbits are caught on the fifth round.

- Exercise: Burpees, squats, jumping jacks.
- Materials: Cones.
- Duration: 10–15 min.

### Appendix B.3. Game 3: The Goldilocks Train

The Goldilocks train is a locomotive that transports the children and childcare workers. The children are walking hand-in-hand in a forward direction acting as the carts of the train. The childcare workers are moving alongside the train carts acting as the moment arms of the train, i.e., the engine of the train.

The children are all walking forward hand-in-hand. The childcare workers runs to the back end of the train and collects a cart (i.e., child) on each side. Then they hold the child hand-in-hand and run to the front of the train. The two children then connects again by holding hands with each other. This sequence continues until the Goldilocks train has reached its destination. Approximately after around 10–15 min.

- Materials: None.
- Duration: 10–15 min.

## Appendix C

**Table A2:** Perceived feasibility of the Goldilocks games sorted by fitness level and smoke status.

**Q1**				
**Fitness Level**	**Workers (*n*)**	**Feasible (%)**	**Partially feasible (%)**	**Unfeasible (%)**
*Above average*	4	83	17	0
*Average*	12	67	33	0
*Below average*	3	89	11	0
**Q2**				
**Fitness level**	**Workers (*n*)**	**Feasible (%)**	**Partially feasible (%)**	**Unfeasible (%)**
*Above average*	4	67	33	0
*Average*	12	75	19	6
*Below average*	3	67	11	22
**Q1**				
**Smoker**	**Workers (*n*)**	**Feasible (%)**	**Partially feasible (%)**	**Unfeasible (%)**
*Yes*	7	62	38	0
*No*	12	81	19	0
**Q2**				
**Smoker**	**Workers (*n*)**	**Feasible (%)**	**Partially feasible (%)**	**Unfeasible (%)**
*Yes*	7	57	43	0
*No*	12	81	8	11

Values are presented with frequency (*n*) or percentage (%). Q1: “To what extent is the game usable in the pedagogical work?”, Q2: “To what extent can you see yourself perform the games in your daily work?”.

## Appendix D

**Table A3:** Borg scale according to smoke status and fitness level.

**Smoker**	**Workers (*n*)**	**Borg (*SD*)**
*Yes*	7	5.0 (1.4)
*No*	12	4.9 (1.5)
**Fitness Level**	**Workers (*n*)**	**Borg (*SD*)**
*Above average*	4	4.6 (1.7)
*Average*	12	4.6 (1.2)
*Below average*	3	6.3 (1.2)

Values are presented with frequency (*n*) or group mean and standard deviation (*SD*).

## References

[1] Margaras V. Demographic Trends in EU Regions. https://ec.europa.eu/futurium/en/system/files/ged/eprs-briefing-633160-demographic-trends-eu-regions-final.pdf.

[2] Lee R., Ferguson R.W., Auerbach A.J., Boersch-Supan A., Bongaarts J., Collins S.M., Lucas C.M., Lucas D.J., Mitchell O.S., Nordhaus W.D. (2012). Aging and the Macroeconomy: Long-Term Implications of an Older Population.

[3] Holtermann A., Mortensen O.S., Burr H., Søgaard K., Gyntelberg F., Suadicani P. (2010). Physical demands at work, physical fitness, and 30-year ischaemic heart disease and all-cause mortality in the Copenhagen Male Study. Scand. J. Work. Environ. Health.

[4] Written K. (2010). Geographies of Obesity.

[5] Buchbinder R., van Tulder M., Öberg B., Costa L.M., Woolf A., Schoene M., Croft P. (2018). Low back pain: A call for action. Lancet.

[6] Burton J. WHO Healthy Workplace Framework and Model: Background and Supporting Literature and Practice. https://www.who.int/occupational_health/healthy_workplace_framework.pdf.

[7] Holtermann A., Coenen P., Krause N., Theorell T. (2020). The paradoxical health effects of occupational versus leisure-time physical activity. Handbook of Socioeconomic Determinants of Occupational Health. Handbook Series in Occupational Health Sciences.

[8] Constitution of the World Health Organization. https://apps.who.int/gb/bd/PDF/bd47/EN/constitution-en.pdf?ua=1.

[9] Warburton D.E., Bredin S.S. (2016). Reflections on physical activity and health: What should we recommend?. Can. J. Cardiol..

[10] Warburton D.E., Nicol C.W., Bredin S.S. (2006). Health benefits of physical activity: The evidence. CMAJ.

[11] Gillen J.B., Martin B.J., MacInnis M.J., Skelly L.E., Tarnopolsky M.A., Gibala M.J. (2016). Twelve weeks of sprint interval training improves indices of cardiometabolic health similar to traditional endurance training despite a five-fold lower exercise volume and time commitment. PLoS ONE.

[12] Holtermann A., Mathiassen S.E., Straker L. (2019). Promoting health and physical capacity during productive work: The Goldilocks Principle. Scand. J. Work. Environ. Health.

[13] Straker L., Mathiassen S.E., Holtermann A. (2018). The ‘Goldilocks Principle’: Designing physical activity at work to be ‘just right’ for promoting health. Br. J. Sports Med..

[14] Mathiassen S.E. (2006). Diversity and variation in biomechanical exposure: What is it, and why would we like to know?. Appl. Ergon..

[15] Straker L., Mathiassen S.E. (2009). Increased physical work loads in modern work—A necessity for better health and performance?. Ergonomics.

[16] Tucker P. (2003). The impact of rest breaks upon accident risk, fatigue and performance: A review. Work Stress.

[17] Jørgensen M.B., Gupta N., Korshøj M., Lagersted-Olsen J., Villumsen M., Mortensen O.S., Skotte J., Søgaard K., Madeleine P., Samani A. (2019). The DPhacto cohort: An overview of technically measured physical activity at work and leisure in blue-collar sectors for practitioners and researchers. Appl. Ergon..

[18] Cumming T. (2016). Early childhood educators’ well-being: An updated review of the literature. Early Child Educ. J..

[19] Linnan L., Arandia G., Bateman L.A., Vaughn A., Smith N., Ward D. (2017). The health and working conditions of women employed in child care. Int. J. Environ. Res. Public Health.

[20] Otten J.J., Bradford V.A., Stover B., Hill H.D., Osborne C., Getts K., Seixas N. (2019). The culture of health in early care and education: Workers’ wages, health, and job characteristics. Health Aff..

[21] Tal og Fakta om Arbejdsmiljøet. https://arbejdsmiljodata.nfa.dk/.

[22] Ward D.S., Vaughn A.E., Hales D., Viera A.J., Gizlice Z., Bateman L.A., Grummon A.H., Arandia G., Linnan L.A. (2018). Workplace health and safety intervention for child care staff: Rationale, design, and baseline results from the CARE cluster randomized control trial. Contemp. Clin. Trials.

[23] Holtermann A., Hendriksen P.F., Schmidt K.G., Svendsen M.J., Rasmussen C.D.N. (2020). Physical work demands of childcare workers in Denmark: Device-based measurements and workplace observations among 199 childcare workers from 16 day nurseries. Ann. Work Expo. Health.

[24] National Reforms in Early Childhood Education and Care. https://eacea.ec.europa.eu/national-policies/eurydice/content/national-reforms-early-childhood-education-and-care-18_en.

[25] The Strengthened Pedagogical Curriculum Framework and Content. https://emu.dk/sites/default/files/2020-01/Den%20styrkede%20pædagogiske%20læreplan_engelsk.pdf.

[26] Kusma B., Mache S., Quarcoo D., Nienhaus A., Groneberg D.A. (2011). Educators’ working conditions in a day care centre on ownership of a non-profit organization. J. Occup. Med. Toxicol..

[27] Adamo K.B., Wasenius N.S., Grattan K.P., Harvey A.L.J., Naylor P.-J., Barrowman N.J., Goldfield G.S. (2017). Effects of a preschool intervention on physical activity and body composition. J. Pediatr..

[28] Tandon P.S., Downing K.L., Saelens B.E., Christakis D.A. (2019). Two approaches to increase physical activity for preschool children in child care centers: A matched-pair cluster-randomized trial. Int. J. Environ. Res. Public Health.

[29] Tucker P., Vanderloo L.M., Johnson A.M., Burke S.M., Irwin J.D., Gaston A., Driediger M., Timmons B.W. (2017). Impact of the supporting physical activity in the childcare environment (SPACE) intervention on preschoolers’ physical activity levels and sedentary time: A single-blind cluster randomized controlled trial. Int. J. Behav. Nutr. Phys. Act..

[30] Brazendale K., Chandler J.L., Beets M.W., Weaver R.G., Beighle A., Huberty J.L., Moore J.B. (2015). Maximizing children’s physical activity using the LET US Play principles. Prev. Med..

[31] Cheung P. (2020). Teachers as role models for physical activity: Are preschool children more active when their teachers are active?. Sage J..

[32] Moher D., Hopewell S., Schulz K., Montori V., Gøtzsche P., Devereaux P., Elbourne D., Egger M., Altman D. (2010). CONSORT 2010 explanation and elaboration: Updated guidelines for reporting parralel group randomised trials. J. Clin. Epidemiol..

[33] Hoffmann T., Glasziou P., Boutron I., Milne R., Perera P., Moher D., Altman D., Barbour V., Macdonald H., Johnston M. (2014). Better reporting of interventions: Template for intervention description and replication (TIDieR) checklist and guide. BMJ.

[34] Van Zandvoort M., Tucker P., Irwin J.D., Burke S.M. (2010). Physical activity at daycare: Issues, challenges and perspectives. Early Years.

[35] Gagné C., Harnois I. (2014). How to motivate childcare workers to engage preschoolers in physical activity. J. Phys. Act. Health.

[36] Björkman F., Ekblom-Bak E., Ekblom Ö., Ekblom B. (2016). Validity of the revised Ekblom Bak cycle ergometer test in adults. Eur. J. Appl. Physiol..

[37] Ekblom-Bak E., Björkman F., Hellenius M.L., Ekblom B. (2014). A new submaximal cycle ergometer test for prediction of VO2max. Scand. J. Med. Sci. Sports.

[38] Hettiarachchi I.T., Hanoun S., Nahavandi D., Nahavandi S. (2019). Validation of Polar OH1 optical heart rate sensor for moderate and high intensity physical activities. PLoS ONE.

[39] Schubert M.M., Clark A., De La Rosa A.B. (2018). The Polar^®^ OH1 optical heart rate sensor is valid during moderate-vigorous exercise. Sports Med. Int. Open.

[40] Maximal oxygen uptake reference values for the Ekblom Bak test. https://www.gih.se/Global/3_forskning/fysiologi/elinekblombak/Reference_values_180319_en.pdf.

[41] Williams N. (2017). The borg rating of perceived exertion (rpe) scale. Occup. Med..

[42] Kristiansen J., Korshøj M., Skotte J.H., Jespersen T., Søgaard K., Mortensen O.S., Holtermann A. (2011). Comparison of two systems for long-term heart rate variability monitoring in free-living conditions—A pilot study. Biomed. Eng. Online.

[43] Skotte J., Korshøj M., Kristiansen J., Hanisch C., Holtermann A. (2014). Detection of physical activity types using triaxial accelerometers. J. Phys. Act. Health.

[44] Korshøj M., Krustrup P., Jespersen T., Søgaard K., Skotte J.H., Holtermann A. (2013). A 24-h assessment of physical activity and cardio-respiratory fitness among female hospital cleaners: A pilot study. Ergonomics.

[45] Tanaka H., Monahan K.D., Seals D.R. (2001). Age-predicted maximal heart rate revisited. J. Am. Coll. Cardiol..

[46] Gormley S.E., Swain D.P., High R., Spina R.J., Dowling E.A., Kotipalli U.S., Gandrakota R. (2008). Effect of intensity of aerobic training on VO2max. Med. Sci. Sports Exerc..

[47] Bacon A.P., Carter R.E., Ogle E.A., Joyner M.J. (2013). VO2max trainability and high intensity interval training in humans: A meta-analysis. PLoS ONE.

[48] Whitley E., Ball J. (2002). Statistics review 4: Sample size calculations. Crit. Care.

[49] Powell K.E., Paluch A.E., Blair S.N. (2011). Physical activity for health: What kind? How much? How intense? On top of what?. Annu. Rev. Public Health.

[50] Warburton D.E.R., Bredin S.S.D. (2017). Health benefits of physical activity: A systematic review of current systematic reviews. Curr. Opin. Cardiol..

[51] R-Core-Team R: A Language and Environment for Statistical Computing. https://www.R-project.org/.

[52] Gupta N., Rasmussen C.L., Holtermann A., Mathiassen S.E. (2020). Time-based data in occupational studies: The whys, the hows, and some remaining challenges in compositional data analysis (CoDA). Ann. Work Expo. Health.

[53] Van den Boogaart K., Tolosana-Delgado R. (2008). “Compositions”: A unified R package to analyze compositional data. Comput. Geosci. UK.

[54] Aitchison J. (1982). The statistical analysis of compositional data. J. R. Stat. Soc. Ser. B Methodol..

[55] Egozcue J.J., Pawlowsky-Glahn V., Mateu-Figueras G., Barceló-Vidal C. (2003). Isometric logratio transformations for compositional data analysis. Math. Geosci..

[56] Pedisic Z., Dumuid D., Olds T. (2017). Integrating sleep, sedentary behaviour, and physical activity research in the emerging field of time-use epidemiology: Definitions, concepts, statistical methods, theoretical framework, and future directions. Kinesiology.

[57] Palarea-Albaladejo J., Martín-Fernández J.Z. (2015). Compositions-R package for multivariate imputation of left-censored data under a compositional approach. Chemometr. Intell. Lab..

[58] Palarea-Albaladejo J., Martín-Fernández J.A. (2008). A modified EM alr-algorithm for replacing rounded zeros in compositional data sets. Comput. Geosci. UK.

[59] Rasmussen C.D.N., Sørensen O.H., van der Beek A.J., Holtermann A. (2020). The effect of training for a participatory ergonomic intervention on physical exertion and musculoskeletal pain among childcare workers (the TOY-project)—a wait-list cluster-randomized controlled trial. Scand. J. Work. Environ. Health.

[60] Jenkins E.M., Nairn L.N., Skelly L.E., Little J.P., Gibala M.J. (2019). Do stair climbing exercise “snacks” improve cardiorespiratory fitness?. Appl. Physiol. Nutr. Metab..

[61] Ramos J.S., Dalleck L.C., Tjonna A.E., Beetham K.S., Coombes J.S. (2015). The impact of high-intensity interval training versus moderate-intensity continuous training on vascular function: A systematic review and meta-analysis. Sports Med..

[62] Tarp J., Child A., White T., Westgate K., Bugge A., Grøntved A., Wedderkopp N., Andersen L.B., Cardon G., Davey R. (2018). Physical activity intensity, bout-duration, and cardiometabolic risk markers in children and adolescents. Int. J. Obes..

[63] Krustrup P., Aagaard P., Nybo L., Petersen J., Mohr M., Bangsbo J. (2010). Recreational football as a health promoting activity: A topical review. Scand. J. Med. Sci. Sports.

[64] Aadahl M., Kjaer M., Jørgensen T. (2007). Perceived exertion of physical activity: Negative association with self-rated fitness. Scand. J. Public Health.

[65] Wells R., Norman R., Frazer M., Laing A., Cole D., Kerr M. (2003). Participative Ergonomic Blueprint.

[66] Parak J., Korhonen I. Accuracy of Firstbeat Bodyguard 2 Beat-To-Beat Heart Rate Monitor. https://assets.firstbeat.com/firstbeat/uploads/2015/10/white_paper_bodyguard2_final.pdf.

[67] Mann T., Lamberts R.P., Lambert M.I. (2013). Methods of prescribing relative exercise intensity: Physiological and practical considerations. Sports Med..

[68] Lidegaard M., Lerche A.F., Munch P.K., Schmidt K.G., Rasmussen C.L., Rasmussen C.D.N., Mathiassen S.E., Straker L., Holtermann A. (2020). Can childcare work be designed to promote moderate and vigorous physical activity, cardiorespiratory fitness and health? Study protocol for the Goldilocks-childcare randomised controlled trial. BMC Public Health.

[69] Linnan L.A., Vaughn A.E., Smith F.T., Westgate P., Hales D., Arandia G., Neshteruk C., Willis E., Ward D.S. (2020). Results of caring and reaching for health (CARE): A cluster-randomized controlled trial assessing a worksite wellness intervention for child care staff. Int. J. Behav. Nutr. Phys. Act..

[70] Proper K.I., van den Heuvel S.G., de Vroome E.M., Hildebrandt V.H., van der Beek A.J. (2006). Dose-response relation between physical activity and sick leave. Br. J. Sports Med..

[71] Jørgensen M.B., Villadsen E., Burr H., Mortensen O.S., Holtermann A. (2015). Does workplace health promotion in Denmark reach relevant target groups?. Health Promot. Int..

